# Changes in the body composition of boys aged 11–18 years due to COVID-19 measures in the Czech Republic

**DOI:** 10.1186/s12889-022-14605-8

**Published:** 2022-12-03

**Authors:** P. Kutac, V. Bunc, M. Sigmund, M. Buzga, M. Krajcigr

**Affiliations:** 1 Department of Human Movement Studies, University of Ostrava, 701 03 Ostrava, Czech Republic; 2 Faculty of Physical Education and Sport, Charles University, Praha 6, 162 52 Praha, Czech Republic; 3 Application Centre BALUO, Faculty of Physical Culture, Palacky University, 779 00 Olomouc, Czech Republic

**Keywords:** Physical activity restriction, Body mass, Body fat, Visceral fat, Fat-free mass, Skeletal muscle mass

## Abstract

**Background:**

The lockdown measures related to coronavirus disease 2019 (COVID) impacted the health of adolescents by reducing physical activity (PA). The physical changes in response to decreases in PA can be measured with full body composition analysis. The aim of this study was to evaluate the effects of long-term PA restrictions on body fat (BF), fat-free mass (FFM) and skeletal muscle mass (SMM) in adolescents.

**Methods:**

A total of 1669 boys (before PA restriction (G1): 998; after PA restrictions ended (G2): 671; between the ages of 11 and 18 were included. The measured parameters were body mass (BM), visceral fat area (VFA), BF, FFM and SMM. The whole-body composition was evaluated using bioelectrical impedance analysis (BIA).

**Results:**

Compared to G1, G2 exhibited an increase in BF between 1.2 and 5.1%. This difference was significant in boys aged 13 to 18 years (*p* < 0.05). VFA increased between 5.3 and 20.5 cm^2^; this increase was significant in boys aged 13 to 18 years (*p* < 0.05). SMM decreased between 2.6 and 3.8%, and this decrease was significant in all age groups (*p* < 0.05). Changes in body composition were not accompanied by any significant changes in BM.

**Conclusions:**

COVID-19 restrictions reduced PA, resulting in a significant decrease in SMM. This decrease may impact boys’ ability to engage in sufficiently varied PA, which may lead to a further decline in PA and subsequent medical consequences in adulthood.

## Introduction

Coronavirus disease 2019 (COVID-19) was officially declared a global health emergency by the World Health Organization (WHO) in January 30, 2020; by July 30, 2022, over 500 million people worldwide had been affected and over 6 million people died [69]. During this period, the WHO provided recommendations for altering public interactions in schools and other public places. Governments followed these recommendations and implemented restrictions varying in severity to stop the spread of COVID-19 [2, 14, 17, 18, 43, 57]. These approaches reduced individual mobility, daily activities [53, 54], and social interactions [58]. As a result, people exhibited higher rates of psychological distress, depression, anxiety, negative feelings, emotional exhaustion, somatic symptoms, and panic disorders [53, 54]. Physical activity (PA), which improves overall health [15, 53, 54, 58], was also significantly restricted [15–17, 44, 45, 53, 54, 58].

Due to the accelerated spread of the disease in the Czech Republic, the government ordered the closure of elementary and secondary schools from March 2020 to May 2020 and from October 2020 to May 2021, for a total of 11 months. Along with school closures (and the introduction of online learning), adolescent leisure activities (including sports clubs organized by schools and sports training) were also halted. School physical education (SPE) lessons were also banned during the short intervals when various forms of in-person education (such as the rotation model of learning) were introduced. Unfortunately, for a substantial proportion of adolescents over the age of ten, SPE constitutes the only regular PA. Moreover, adolescents engage in less PA as their age increases [6, 20]. Childhood and early adolescence are considered sensitive periods for the development of physical literacy [36], which is deemed the foundation for lifelong health and fitness [64, 65], and the lack of PA causes considerable changes in overall health [15, 53, 54, 58]. Therefore, mandatory SPE represents a crucial part of PA. These lessons not only ensure the mandatory participation of adolescents in PA but also improve physical fitness (cardiorespiratory fitness and aerobic fitness) and reduce the risk of future cardiovascular diseases [49]. The physical fitness of adolescents is closely correlated with the duration of SPE and with an active lifestyle [11]. Systematic reviews evaluating the impact of regular PA on medical indicators in adolescents have reported that sports and PA are favourably related to indicators of physical, psychological, social, and cognitive health. These indicators include cardiometabolic markers, adiposity, bone and musculoskeletal health, physical fitness, and the development of motor skills [8, 46, 48]. Additionally, engaging in sports as a youth increases the amount of regular PA in adulthood [29].

A sedentary lifestyle, the current predominant lifestyle, negatively affects the physical development of adolescents and reduces their ability to engage in various forms of PA [3]. These limited motor competencies are both a cause and a consequence of health and social pathologies. A sedentary lifestyle leads to a considerable increase in overweight and obesity as well as reduced fitness. Therefore, the implementation of PA in the child population is urgently needed [7, 10, 19]. One of the methods to evaluate the general health, nutritional condition, and overall fitness of an individual is to analyse their body composition [1, 37]. The body composition of adolescents reflects their behaviour and may impact their current condition [52]. Body composition is influenced by both genetic and exogenous factors, including overall health, nutrition, and PA. Studies have documented the effect of PA on body composition [35, 50].

A reduction in protein intake likely leads to a reduction in skeletal muscle mass (SMM). A 2022 meta-analysis by Córdoba-Rodriguez proposed connections of lower SMM in adolescents with metabolic syndrome and the related impairments in glucose tolerance and insulin resistance [13]. Studies of adults have shown that the proportion of fat-free mass (FFM) decreases according to increases in body fat (BF), while the overall body mass index (BMI) remains the same [21, 33]. It is highly likely that the same phenomenon occurs in adolescents, especially in those with impaired glucose tolerance, insulin resistance, or metabolic syndrome [5]. Ultimately, this condition leads to qualitative changes in muscle mass without changes in the overall body weight of the individual. The connection between fat tissue hypertrophy and insulin resistance described in the European multicentric study, HELEN, is a serious issue [26, 51].

The following working hypothesis was developed based on recent evidence regarding the association between PA and body composition: the restrictions in PA implemented during the COVID-19 pandemic will lead to a significant increase in body mass (BM) and BF and a significant decrease in FFM and SMM in adolescent boys.

Implementation of PA restrictions in schools was determined by the Czech Ministry of Health without considering the impact of such measures beyond protection from COVID-19. Therefore, the objective of this study was to assess the impact of decreased PA due to COVID-19 restrictions in adolescents using body composition analysis.

## Materials and methods

### Participants

A total of 1669 boys (aged 11 to 18 years) took part in the study. Of these boys, 998 were assessed before the PA restrictions (G1), and 671 were assessed after the PA restrictions due to COVID-19 were lifted (G2). Girls are more prone to changes related to physiological processes (such as menstruation) than boys; thus, they were excluded from the study. Purposive sampling was employed to select from the available population of North Moravian males within the desired age group. Approximately 35% of participants were measured twice, once before the PA restrictions (G1) and once after the PA restrictions were lifted (G2). We were unable to include more subjects in the study due to the lack of cooperation from school management and parents. Some adolescents from the first assessment did not take part in the second assessment (G2) due to a positive COVID-19 test; however, because we initially recruited a large sample size, we believe that the study results still provide useful data. We assessed the current developmental stage of boys (i.e., occurrence of puberty) according to the methods of Mirwald et al. [40] and Müller et al. [42]. We also classified the participants by chronological age. The number of participants in each age group is presented in Table 1. Age classification was performed according to the WHO classification, where individuals are classified into age categories after reaching the chronological age (e.g., 6 years old = 6.00–6.99 years old) [61]. All the recorded adolescents were Caucasian. The inclusion criteria were as follows: no objective medical conditions based on a medical check by a paediatrician, chronological age of 11-18 years and no regular PA outside the school (to exclude adolescents with specific sports training). Minors were informed of the study objectives and were able to refuse to participate despite parental provision of informed consent. The adolescents engaged in mandatory SPE twice per week, with each session lasting 45 min. All the participants voluntarily took part in the research; they were informed about the measurement procedure in advance, and their parents or legal representatives signed an informed consent form to participate in the study. Boys that were 18 years old provided informed consent (i.e., without needing a parent or guardian). The research was approved by the Ethics Committee of Palacký University (No. 76/2016) and the Faculty of Education at the University of Ostrava (No. OU-22702/45-2022). The study procedure and methods were in compliance with the 1964 Helsinki Declaration and its later amendments or comparable ethical standards. Neither the participants nor their legal guardians received any incentives to ensure their participation in the study. The study was conducted during a nonprofit event.

**Table 1:** Participants in the measured age categories

Age (years)	11	12	13	14	15	16	17	18
G1	103	86	121	128	121	134	144	161
G2	111	66	52	61	62	109	121	89
Σ	214	152	173	189	183	243	265	250

G1 – measurement before PA restriction, G2 – measurement after PA resumed, Σ - sum

### Procedures

**Fig. 1:**
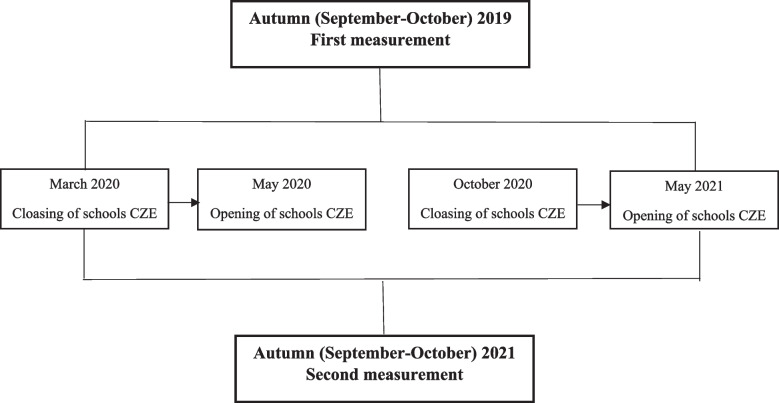
Flow chart of the study process

G1 measurements were collected from September to October 2019, and G2 measurement were collected from September to October 2021. The study protocol is presented in Fig. 1. The measurements were collected in the medical rooms of each school, which are designated to treat potential student injuries (especially during physical education (PE) classes). The cross-sectional measurements were collected once from each participant. These measurements were recorded by the same research team under the supervision of the first author using the same method and analyser and at the same time of the day (between 8 and 10 a.m.). The assessment of body mass composition and other anthropometric parameters occurred in the morning and observed the measurement principles stated in a professional study on the issues of using bioelectrical impedance analysis (BIA) [39]. Because BIA measures body fluid volumes as a basic parameter and the current state of body hydration depends on fluid intake, it is necessary to control fluid intake before measurement [55]. Therefore, the participants (or the parents of minors) received precise instructions to avoid intense PA (for 24 h prior to measurement), food and drink (4 h prior to measurement) and caffeine (12 h prior to measurement). These recommendations were based on the recommendations in the aforementioned study [39]. The guardians of minors and adult participants also received individual recommendations for drinking prior to measurement to ensure hydration in the measured adolescents based on their BM and PA as well as the season. Adherence to these recommendations was verified by questions just before using the BIA analyser. The adolescents were measured in sports clothing (shorts and a T-shirt) and were barefoot.

The measured parameters were body height (BH), total BM and body composition parameters: absolute and relative BF, visceral fat area (VFA; in cm^2^), FFM, and absolute and relative SMM. BH was measured using a Tanita HR 001 stadiometer (Tanita, Japan) with precision of 0.5 cm. BM and body composition were measured by a tetrapolar bioimpedance multifrequency InBody 770 analyser (Biospace, South Korea) according to the recommendations of the manufacturer. The parameters measured in kilograms (BM, BF, and FFM) were measured to the nearest 0.1 kg, the parameters measured in percentage (BF and SMM) were measured to the nearest 0.5%, and VFA was measured to the nearest 0.5 cm^2^. The InBody 770 analyser was selected due to its worldwide use in diagnostic practice. BMI was calculated from BH and BM. The sixth national anthropological study of the Czech Republic (6th NAS) percentile growth chart [60] was used for group classification and BMI analysis. BMI was categorized according to percentiles: P1 (under the 25th percentile), thin; P2 (25–75th percentile), proportionate; P3 (75–90th percentile), plump; P4 (90–97th percentile), overweight; and P5 (over the 97th percentile), obese. Based on BMI and the current chronological age, participants were registered in the corresponding percentile range of the 6th NAS growth chart, which is the current Czech population standard [60].

### Data analysis

According to the results of the Shapiro–Wilk test, all monitored parameters had a normal distribution. To evaluate statistical significance, we used the parametric t test. Practical importance of the statistically significant differences was determined using Cohen’s *d*, an indicator of effect size (ES); *d* = 0.2 indicates a minor effect, *d* = 0.5 indicates a moderate effect, and *d* = 0.8 indicates a large effect [12]. A value of Cohen’s *d* ≥ 0.5 was considered practically important. To compare the representation of boys in the individual percentile bands in the growth charts, we used the chi-square test and the Bonferroni post hoc test, which was computed from the residuals with a modified *p* value of .05/16 = 0.003125 for each classification (P1-P5) [25]. The level of statistical significance for all tests was set at α = 0.05. Statistical analysis was performed with SPSS (version 21 for Windows; IBM, Armonk, NY, USA).

## Results

Data are expressed as means and standard deviations for boys measured before PA restrictions (Table 2) and after PA restrictions were lifted (Table 3). Group differences are shown in Table 4. Table 5 presents the detailed group differences in BMI.

**Table 2:** Mean values of somatic characteristic – measurement before restricted the PA (G1)

Parameters	Age (years)							
	11	12	13	14	15	16	17	18
	M ± SD	M ± SD	M ± SD	M ± SD	M ± SD	M ± SD	M ± SD	M ± SD
BH (cm)	149.1 ± 6.1	154.9 ± 7.3	165.5 ± 9.6	172.7 ± 8.3	177.7 ± 6.7	178.7 ± 6.6	179.8 ± 7.5	180.5 ± 7.0
BM (kg)	42.0 ± 9.2	44.7 ± 9.9	53.6 ± 10.9	61.1 ± 10.3	66.0 ± 8.1	70.1 ± 11.3	72.9 ± 12.2	75.1 ± 10.3
BMI (kg/m^2^)	18.8 ± 3.4	18.5 ± 3.4	19.4 ± 2.6	20.4 ± 2.3	20.9 ± 2.0	22.0 ± 3.3	22.5 ± 3.1	23.0 ± 2.7
BF (kg)	9.6 ± 6.2	7.9 ± 5.4	7.2 ± 5.0	6.1 ± 4.1	6.7 ± 3.6	9.4 ± 6.2	10.1 ± 6.3	10.1 ± 5.6
BF (%)	21.3 ± 9.3	16.5 ± 7.7	13.1 ± 7.2	9.7 ± 5.2	10.0 ± 4.9	12.8 ± 6.6	13.3 ± 5.9	13.1 ± 5.3
VFA (cm^2^)	54.2 ± 32.8	36.8 ± 27.7	30.7 ± 25.9	23.1 ± 20.5	25.9 ± 18.6	38.9 ± 29.4	42.2 ± 28.3	41.2 ± 26.9
FFM (kg)	32.4 ± 4.5	36.8 ± 6.2	46.3 ± 9.0	55.0 ± 8.7	59.3 ± 7.2	60.7 ± 8.0	62.8 ± 8.5	65.0 ± 7.4
SMM (kg)	17.2 ± 2.7	19.8 ± 3.7	25.5 ± 5.4	30.8 ± 5.2	33.4 ± 4.3	34.3 ± 4.8	35.6 ± 5.1	37.0 ± 4.5
SMM (%)	41.6 ± 4.9	44.9 ± 4.1	47.7 ± 4.3	50.5 ± 3.2	50.7 ± 2.7	49.2 ± 3.9	49.1 ± 3.5	49.4 ± 3.1

*BH* body height, *BM* body mass, *BMI* body mass index, *BF* body fat, *VFA* visceral fat area, *FFM* fat free mass, *SMM* skeletal muscle mass, *M* mean, *SD* standard deviation

**Table 3:** Mean values of somatic characteristic – measurement after resumed the PA (G2)

Parameters	Age (years)							
	11	12	13	14	15	16	17	18
	M ± SD	M ± SD	M ± SD	M ± SD	M ± SD	M ± SD	M ± SD	M ± SD
BH (cm)	149.9 ± 6.4	155.7 ± 8.0	163.9 ± 8.8	172.4 ± 8.8	177.7 ± 6.0	178.0 ± 6.1	179.1 ± 7.3	180.1 ± 6.3
BM (kg)	44.3 ± 9.2	45.4 ± 10.5	55.7 ± 12.1	62.7 ± 13.6	65.8 ± 9.0	70.3 ± 10.1	74.2 ± 13.1	75.0 ± 12.4
BMI (kg/m^2^)	19.6 ± 3.4	18.6 ± 3.6	20.6 ± 3.3	21.0 ± 3.7	20.8 ± 2.6	22.2 ± 3.1	23.1 ± 3.3	23.1 ± 3.8
BF (kg)	10.9 ± 6.7	8.6 ± 6.0	10.5 ± 7.5	9.9 ± 7.1	8.7 ± 5.0	11.1 ± 6.3	12.4 ± 7.1±	13.2 ± 8.2
BF (%)	23.1 ± 9.6	17.7 ± 7.9	17.9 ± 8.9	14.8 ± 7.6	12.8 ± 5.6	15.3 ± 6.6	16.0 ± 6.3	16.8 ± 7.4
VFA (cm^2^)	59.5 ± 35.0	42.2 ± 29.9	47.5 ± 35.8	43.6 ± 34.1	38.2 ± 24.4	47.4 ± 28.9	53.4 ± 30.4	56.4 ± 36.6
FFM (kg)	33.4 ± 4.7	36.8 ± 6.6	45.2 ± 8.1	52.9 ± 9.3	57.1 ± 6.2	59.1 ± 7.0	61.8 ± 8.3	61.8 ± 7.5
SMM (kg)	16.8 ± 3.1	19.1 ± 4.2	24.3 ± 4.9	29.0 ± 5.6	31.5 ± 3.7	32.6 ± 4.3	34.2 ± 5.1	34.5 ± 4.5
SMM (%)	38.6 ± 5.1	42.3 ± 4.5	43.9 ± 5.3	46.7 ± 4.4	48.0 ± 3.3	46.6 ± 3.7	46.4 ± 3.5	46.4 ± 4.3

*BH* body height, *BM* body mass, *BMI* body mass index, *BF* body fat, *VFA* visceral fat area, *FFM* fat free mass, *SMM* skeletal muscle mass, *M* mean, *SD* standard deviation

**Table 4:** Differences in the mean values of somatic parameters of boys measured after resumed the PA and before restricted the PA (G2 versus G1) – statistical analysis results

Parameters	Age (years)							
	11	12	13	14	15	16	17	18
	Diff	Diff	Diff	Diff	Diff	Diff	Diff	Diff
BH (cm)	+ 0.8	+ 0.8	−1.6	−0.3	0	−0.7	−0.7	−0.4
BM (kg)	+ 2.3	+ 0.7	+ 2.1	+ 1.6	−0.2	+ 0.2	+ 1.3	−0.1
BMI (kg/m^2^)	+ 0.8	+ 0.1	+ 1.2*^+^	+ 0.6	− 0.1	+ 0.2	+ 0.6	+ 0.1
BF (kg)	+ 1.3	+ 0.7	+ 3.3**^++^	+ 3.8***^++^	+ 2.0**^++^	+ 1.7*^+^	+ 2.3**^+^	+ 3.1**^+^
BF (%)	+ 1.8	+ 1.2	+ 4.8***^++^	+ 5.1***^+++^	+ 2.8***^++^	+ 2.5**^+^	+ 2.7***^+^	+ 3.7***^++^
VFA (cm^2^)	+ 5.3	+ 5.4	+ 16.8**^++^	+ 20.5***^+++^	+ 12.3***^++^	+ 8.5*^+^	+ 11.2**^+^	+ 15.2***^++^
FFM (kg)	+ 1.0	0	−1.1	−2.1	−2.2*^+^	−1.6	− 1.0	−3.2**^+^
SMM (kg)	−0.4	+ 0.7	−1.2	− 1.8*^+^	− 1.9**^+^	−1.7**^+^	− 1.4*^+^	−2.5***^++^
SMM (%)	−3.0***^++^	−2.6***^++^	−3.8***^+++^	− 3.8***^+++^	− 2.7***^+++^	− 2.6***^++^	−2.7***^++^	−3.0***^+++^

*BH* body height, *BM* body mass, *BMI* body mass index, *BF* body fat, *VFA* visceral fat area, *FFM* fat free mass, *SMM* skeletal muscle mass, *Diff* difference

^*^p < 0.05

^**^p < 0.001

^***^p < 0.0001

^+^*d* < 0.5

^++^*d* ≥ 0.5

^+++^*d* ≥ 0.8

**Table 5:** Frequency of representation of G1 and G2 boys in the percentile bands of the 6th NAS growth chart

Age (years)	P1 (p < 25)		P2 (p 25-75)		P3 (p 75-90)		P4 (p 90-97)		P5 (p > 97)	
	G1 n (%)	G2 n (%)	G1 n (%)	G2 n (%)	G1 n (%)	G2 n (%)	G1 n (%)	G2 n (%)	G1 n (%)	G2 n (%)
11	23(22.3)	7(6.3)	**38(36.9)**	**51(45.9)**	19(18.4)	21(18.9)	10(9.7)	17(15.3)	13(12.6)	15(13.5)
12	29(33.7)	18(27.3)	32(37.2)	32(48.5)	11(12.8)	6(9.1)	10(11.6)	5(7.6)	4(4.7)	5(7.6)
13	18(14.9)	3(5.8)	67(55.4)	28(53.8)	18(14.9)	11(21.2)	11(9.1)	4(7.7)	7(5.8)	6(11.5)
14	11(8.6)	10(16.4)	76(59.4)	23(37.7)	21(16.4)	12(19.7)	15(11.7)	11(18.0)	5(3.9)	5(8.2)
15	13(10.7)	12(19.4)	67(55.4)	26(41.9)	30(24.8)	16(25.8)	8(6.6)	6(9.7)	3(2.5)	2(3.2)
16	23(17.2)	17(15.6)	66(49.3)	41(37.6)	21(15.7)	31(28.4)	10(7.5)	10(9.2)	14(10.4)	10(9.2)
17	23(16.0)	14(11.6)	70(48.6)	52(43.0)	23(16.0)	29(24.0)	13(9.0)	12(9.9)	15(10.4)	14(11.6)
18	18(11.2)	22(24.7)	79(49.1)	27(30.3)	41(25.5)	19(21.3)	16(9.9)	12(13.5)	7(4.3)	9(10.1)
p value	0.04		5.32 × 10^−7^		0.02		0.44		0.98	

n – frequency, G1 – boys measured before anti-pandemic measures, G2 – boys measured after the end of anti-pandemic measures

No significant group differences in BH were observed. Similarly, no significant group differences in BM were found, even though there was a nonsignificant increase in G2 compared to G1, except for 15-year-old and 18-year-old boys. This increase was reflected in the lack of significant group differences in BMI, except for among 13-year-old boys who exhibited a significant increase. However, this increase did not meet the threshold for practical importance (*d* < 0.5).

An increase in the BF parameters, both in the absolute (kilograms) and relative (percentage) values, was observed in all age groups of G2 boys. Statistically significant differences were found in 13–18-year-old boys (*p* < 0.05); however, practical importance (*d* ≥ 0.5) was only confirmed in the BF (kg) of boys between the ages of 13 and 15 years and in the BF (%) of boys 13–15 years and 18 years of age. Similar results were observed in terms of VFA.

The G2 boys showed a decrease in FFM and SMM (except for 11-year-old and 12-year-old boys). However, the only decrease that was both statistically and practically significant (*d* ≥ 0.5) was in the SMM (kg) (Table 4) of 18-year-old boys. In all age groups, a significant decrease in the percentage of SMM was observed in G2 boys, with a statistical significance of *p* < 0.05 and a practical importance of *d* = 0.55–1.05.

All the differences reported exceeded the typical error of measurement of such parameters on the BIA analyser used [32].

Each boy was classified according to the corresponding percentile band in the growth chart. G1 and G2 were compared using the chi-squared test and Bonferroni post hoc test. The *p* value of the chi-square test is described for the bands of the percentile classifications (P1-P5). Statistically significant values are highlighted in Table 4. No significant group differences were observed in the representation of the percentile bands, except that 11-year-old G2 boys had a higher representation in P2. This difference was accompanied by a higher representation of G1 adolescents in P1 and lower representation in P3, P4 and P5. However, these differences were not significant. The results correspond with the overall BMI evaluation (Table 4).

## Discussion

Our hypotheses that the school PA restrictions would lead to a significant increase in BM and BF and a significant decrease in FFM and SMM (as represented by differences between the G1 and G2 boys) were only partially confirmed. There was no significant increase in BM in the G2 group; however, there was a significant increase in absolute BF (in kg) in 13–15-year-old G2 boys and in relative BF (%) in G2 boys aged 13–15 and 18 years. There was no significant decrease in FFM in G2 boys. However, there was a decrease in absolute SMM (in kg) in 18-year-old G2 boys, and the relative SMM (%) decreased in G2 boys in all age categories. A strength of this study is that we used only objective somatic parameters rather than subjective cognitive instruments (tests, questionnaires, etc.).

Creating conditions to safely engage in PA of sufficient variety is one of the basic prerequisites for including PA in the daily routine of adolescents to meet the minimum amount recommended by the WHO. At present, the recommended amount of PA for adolescents is 60 minutes of daily moderate to vigorous PA (mostly aerobic) throughout the week and vigorous aerobic PA, as well as PA to strengthen muscle and bone, 3 days a week [68]. Before the COVID-19 pandemic, 80% of adolescents worldwide did not follow the WHO recommendations [22]. The Health Behavior in School-aged Adolescents (HBSC) study reported that only 23.1% of boys and 14.0% of girls between the ages of 13 and 15 years adhered to the WHO’s PA recommendations [67]. In Central European countries (the Visegrad Group), the recommendations were met by 8.0–32.3% of adolescents in the Czech Republic, 8.5-14.6% of adolescents in Hungary, 7.5-69.4% of adolescents in Poland and 25.1% of adolescents in Slovakia [28]. The implementation of COVID-19 measures further reduced the time that adolescents engaged in PA [47]. This decrease was confirmed by a study of the PA of European adolescents in January and February of 2021 that reported that only 9.3% of adolescents met the PA recommendations of the WHO [30]. A higher proportion of sedentary behaviour in adolescents is associated with worse physical fitness and cardiometabolic health as well as increased adiposity [4, 9]. Thus, insufficient PA in childhood increases future health risks, as a physically inactive child is highly likely to become an inactive adult. Physical inactivity, along with hypertension, cholesterol (mainly low-density lipoprotein cholesterol), type 2 diabetes mellitus (T2DM) and high glucose levels, is a risk factor for premature morbidity worldwide [66].

Less physically active adolescents, who have lower physical skills, are also at risk of injuries while engaging in PA (for example, during mandatory PE lessons in school) [71]. These injuries are related to the atrophy of SMM, which is characterized by the weakening, shrinkage, and reduction of muscle mass as well as decreases in the cross-sectional area of muscle fibres. This atrophy manifests as reduced strength, faster fatigue, and reduced motor ability [70]. Muscle atrophy due to insufficient exercise can be caused by illness, recovery from an injury, or insufficient PA [62].

We found significant changes in body composition parameters in G2 boys (assessed after the end of the COVID-19 measures), without significant changes in body weight (Table 4), as reflected in the absence of significant changes in BMI. G2 boys also exhibited an increase in BF; this difference not only exceeded the typical measurement error but also exceeded the daily variation in body composition reported when using BIA analysers [31]. In addition to the increased BF, G2 boys also exhibited an increase in VFA (Table 4), which is a more serious finding. Visceral fat has higher metabolic activity than subcutaneous fat, and thus imposes higher risks [41]. Evidence has confirmed the associations of visceral fat with cardiovascular complications, obesity, and T2DM [23, 56, 59]. Thus, G2 boys exhibited a higher risk of future health issues (e.g., weight gain, T2DM, high blood pressure) due to the reduction in PA.

We also observed a significant difference in SMM; specifically, all age groups of G2 boys exhibited lower relative SMM (%) than G1 boys. This finding is consistent with recent studies showing that obese adolescents have a lower protein content and a higher fat content at similar or higher BMI values [24].

We believe that the population we examined was affected by the reduction in PA during a highly vulnerable developmental stage in terms of the risks of obesity and metabolic syndrome. A study by Mesinovic et al. [38] of overweight and obese adults and people with metabolic syndrome showed a lower quality of muscle tissue although there was a higher quantity of relative FFM. Similarly, a Korean study showed a lower handgrip strength in adolescents with metabolic syndrome [27]. However, it remains unclear how the physical fitness and body composition of the studied population in adulthood will be affected by reduced PA and muscle mass. Even though our study was purely cross-sectional, combining these results with those of other studies indicates an elevated risk of serious metabolic diseases related to obesity and diabetes due to insufficient adolescent PA. Similarly, the mechanisms linking FFM/SMM with insulin resistance, glucose tolerance, and metabolic syndrome are unknown; however, it appears that a reduction in muscle mass (specifically, in type II fibres) leads to reduced fibre capillarization, numbers of mitochondria, and, ultimately, utilization of glucose. Conversely, decreases in SMM lead to an increase in the accumulation of intramuscular fat [33, 34]. These pathophysiological mechanisms represent increased risks for metabolic diseases, such as T2DM and metabolic syndrome among child and adolescent populations (once they reach adulthood) [63].

## Limitations of the study

This study has several limitations. The main limitation in our study is the lack of data on the pubertal stage of our population. Indeed, the difference in the pubertal stage of the participants and their respective anabolism (mainly testosterone secretion) would bias our results. The second limitation arises from the size of the G2 sample. The G2 boys were measured in an interval between COVID-19 waves; therefore, it was more difficult to obtain the consent of school management and guardians for student participation. The third limitation is that this was a cross-sectional study; thus, we proposed a relation between the PA restrictions at schools and body composition but were unable to establish causality. The fourth limitation concerns the use of the 6th NAS growth chart to evaluate BMI. The growth chart was designed based on measurements in 2001, but there are no current growth charts for Czech adolescents. The fifth limitation includes the absence of a nutritional or caloric intake assessment, as eating habits are a key factor that may influence body composition, namely, the fat component. Unfortunately, we were unable to accurately assess this parameter, as there are no up-to-date population standards. Future studies should recruit other Czech participants of the same age, which may increase the sample size and reproducibility of the results, as they are currently valid only in the North Moravian area.

## Conclusions

After the PA restriction (cessation of PE and sports club activities) at schools, we found a significant decrease in SMM in all age categories of the monitored boys. This decrease has serious consequences, as a reduction in SMM may prevent boys from engaging in a sufficient variety of PA in the future, which may further reduce the amount of PA (and increase the risks of all associated medical consequences) in adulthood. Other negative qualitative changes in body mass may occur, further decreasing the likelihood of regular engagement in PA.

We observed no significant changes in BM; however, we found negative changes in the quality of BM that should be considered when implementing PA restrictions in the future. Such measures should not prevent or reduce PA for adolescents at schools. Sufficient conditions for engaging in PA should be preserved within schools (e.g., PE and sports club activities).

## Data Availability

All data generated or analysed during this study are included in this published article.
